# Time-related improvement of survival in resectable gastric cancer: the role of Japanese-style gastrectomy with D2 lymphadenectomy and adjuvant chemotherapy

**DOI:** 10.1186/1477-7819-4-53

**Published:** 2006-08-11

**Authors:** Juan J Grau, Ramon Palmero, Maribel Marmol, Jose Domingo-Domenech, Mariano Monzo, Jose Fuster, Oscar Vidal, Constantino Fondevila, Juan C Garcia-Valdecasas

**Affiliations:** 1 Oncology Department, ICMHO (Institut Clinic de Malalties Hemato-Oncologiques) and IDIBAPS (Instituto de Investigaciones Biomédicas Augusto Pi Sunyer); University of Barcelona. Hospital Clinic, Barcelona, Spain; 2 Gastrointestinal Surgery Depart. University of Barcelona. Hospital Clinic, Barcelona, Spain

## Abstract

**Background:**

We investigated the change of prognosis in resected gastric cancer (RGC) patients and the role of radical surgery and adjuvant chemotherapy.

**Methods:**

We retrospectively analyze the outcome of 426 consecutive patients from 1975 to 2002, divided into 2 time-periods (TP) cohort: Before 1990 (TP1, n = 207) and 1990 or after (TP2; n= 219). Partial gastrectomy and D1-lymphadenetomy was predominant in TP1 and total gastrectomy with D2-lymphadenectomy it was in TP2. Adjuvant chemotherapy consisted of mitomycin C (MMC), 10–20 mg/m2 iv 4 courses or MMC plus Tegafur 500 mg/m2 for 6 months.

**Results:**

Positive nodes were similar in TP2/TP1 patients with 56%/59% respectively. Total gastrectomy was done in 56%/45% of TP2/TP1 respectively. Two-drug adjuvant chemotherapy was administered in 65%/18% of TP2/TP1 respectively. Survival at 5 years was 66% for TP2 versus 42% for TP1 patients (p < 0.0001). Survival by stages II, IIIA y IIIB for TP2 versus TP1 patients was 70 vs. 51% (p = 0.0132); 57 vs. 22% (p = 0.0008) y 30 vs. 15% (p = 0.2315) respectively. Multivariate analysis showed that age, stage of disease and period of treatment were independent variables.

**Conclusion:**

The global prognosis and that of some stages have improved in recent years with case RGC patients treated with surgery and adjuvant chemotherapy.

## Background

For many authors, gastric carcinoma remains one of the leading causes of cancer death worldwide, second only to lung carcinoma [1,2]. Five-year relative survival of patients from European countries ranges from 10 to 30% [3,4], similar to that reported in USA (15 to 28%) [5]. Local and regional gastric carcinoma showed a 5-year relative survival of 55–59% and 20–22% respectively [6]. In this latter subgroup of patients the surgical treatment of choice consisted of gastrectomy combined with regional lymph node dissection. The relevance of radical surgery, extending lymph node dissection as wide as possible has been highlighted. The experience of an expert surgeon has been shown to improve clinical outcome in some tumors [7]. In the statistical outcome of two European trials, one from the United Kingdom and the other, The Netherlands, comparing D1 versus D2 lymphadenectomy a survival rate of approximately 20% for D1 group was assumed [8,9]. This 20% overall survival was based on historic data from both those countries. Nevertheless, the overall 5-year survival for D1 group jumped to 34% and 45% respectively, without any dramatic change in T classification distribution, suggesting that the results from expert surgeons may improve final cure rates [10,5].

In spite of surgical treatment, thousands of loco-regional gastric cancer patients relapse and die worldwide each year. Taking into account the poor survival of gastric carcinoma after treatment only with surgery, several adjuvant strategies have been developed in recent years to reduce relapse rates and to improve long-term survival. Survival rates of up to 40% in selected patients receiving postoperative adjuvant chemoradiation has been obtained after curative resection (R0) in contrast to 30% survival if patients were treated with surgery alone [11]. Japanese authors have proposed that improvement in survival can be also achieved with surgery plus adjuvant chemotherapy based on mitomycin and fluorouracil derivates [12], thus avoiding toxic effects through radiotherapy. Likewise, our group reported a 60% 5-year survival among patients with loco-regional gastric carcinoma treated with gastrectomy followed by 6 months of chemotherapy based on mitomycin-C and tegafur (a 5-fluoruracil pro-drug) without radiotherapy [13,14].

Our prospectively maintained database contains data on patients with early and locally advanced gastric carcinoma treated with surgery since 1975. After 1990 the principal surgical option was D2 dissection plus gastrectomy. Initially, patients who achieved disease-free status after surgery were offered the option of adjuvant chemotherapy within a clinical trial or follow-up with no further therapy. After 1990, we offered adjuvant chemotherapy to all patients. In order to evaluate the improvement in the prognosis among operated gastric cancer patients, we have retrospectively compared the long-term therapeutic results of patients diagnosed and treated at our institution before and after 1990.

In this study, we analyzed the outcome and survival of resected non-metastatic gastric cancer patients over this time period, comparing the periods before and after 1990 when Japanese-style surgery followed by adjuvant chemotherapy were included as the preferable treatment option for the majority of patients.

## Patients and methods

This retrospective study includes 426 consecutive non-metastatic patients who underwent primary surgery for gastric adenocarcinoma with curative R0 intent (stages Ia to IV M0).

Since 1975, patients with early or locally advanced gastric adenocarcinoma have been operated with subtotal or total gastrectomy according to the location and the size of the primary tumor. The range of lymph node resection was performed following the classification and rules of the Japanese Research Society for the Study of Gastric Cancer [15]. D1 resections were performed until 1990 when, after a 6-months training program in Japan, surgeons standardized the use of the more extended D2 dissection at our institution. Resections of spleen and tail of pancreas were performed in proximal gastric tumors to achieve adequate removal of D2 lymph node stations 10 and 11.

Each patient was staged according to the tumor-node-metastasis system valid at the time of surgery. Recently, the database has been updated and patients diagnosed before 1997 were re-staged according to the latest guidelines of the AJCC published in 1997 [16].

### Adjuvant chemotherapy

From 1975 to 1990, patients after complete resection of gastric cancer were offered adjuvant chemotherapy treatment within clinical trials or they were followed up without treatment. Since 1990, adjuvant chemotherapy has been offered to all patients, both within and outside the framework of clinical trials. Two different chemotherapy schedules were used: Mitomycin-C (MMC) 10–20 mg/m2 i.v. bolus once every 6 weeks, or MMC plus Tegafur (TG) 500 mg/m2 p.o. daily. Both regimens were administered during 6 months after surgery. After 1995, MMC-TG was routinely to all patients out of clinical trials. After adjuvant treatment, all patients were followed up with clinical checking, biochemical blood test, including tumor makers (CEA and CA 19.9), chest X-ray film, and liver ultrasonography every 3 to 6 months for five years and yearly thereafter. Other explorations such as CT-scan or endoscopy were performed if clinical or complementary test alterations appeared.

### Statistical analysis

Follow-up and survival data were recorded according to the rules set down by Peto *et al *[17]. The database was last updated on 30 November 2004. In this study, data were retrospective analyzed. For statistical analysis the SPSS program (version 11.0, SPSS Inc, Chicago) was used. Comparison between groups based on patient's characteristics was performed using the χ^2^test for discrete data and t-test for continuous data, both two-tailed. Patients were divided firstly in two groups, depending on the time period (TP) in which they received treatment i.e. TP1 = 1975–1989, and TP2 = 1990–2002. Secondly, global survival of all patients was divided in five-year period). Survival probabilities were estimated by the Kaplan-Meier product-limit method [18] and the log-rank test was used to evaluate the difference between survival curves [19]. A p-value of 0.05 was considered to be the limit of significance for all analysis.

Cox's proportional hazard model with covariates for main prognostic factors like gender, positive lymph nodes, adjuvant chemotherapy and period of treatment was applied.

## Results

Patient characteristics are outlined in Table 1. In the TP1, 207 patients were included in the study as they were diagnosed and treated for primary gastric cancer, while in TP2, the patients included with the same criteria were 219. There were no significant differences in gender distribution, with a male/female ratio of 2/1. Patients TP2 were older than the TP1 (>61 years: 46% vs. 68%; p = 0,001). Distribution of tumor location was well balanced. The proportion of T1/T2 tumors in TP2 patients was higher compared with those TP1 patients (35% versus 21%, p < 0,001). There were no differences between the two groups in the proportion of node positive/negative staging, (56.1%/43.8% in TP2 patients and 58.9%/41.1% in TP1 patients). Because the staging rules, node positive N3 tumors were observed in 9 out of 219 (4%) (TP2 patients versus none in TP1 patients). In spite of these differences, a similar distribution of stages was seen in both groups, stage I plus II/stage III plus IV in TP2 patients (50.2%/49.8) compared with those TP1 patients (47.4%/52.6%)(p = 0,562).

**Table 1:** Patient's demographics and tumors characteristics

		**Year of diagnostic**				
			
		**Before 1990**		**1990 and after**		
		**(n = 207)**		**(n = 219)**		
			
	**Total**	**%**	**n**	**%**	**n**	**ρ **
Gender						= .919
Male	276	64.9	135	64.4	141	
Female	150	35.1	72	35.6	78	
Age. years						< .001
< 61	182	54.1	112	31.7	70	
> 61	244	45.9	95	68.3	149	
Tumor location*						= .097
Cardia	32	6.3	10	11.6	22	
Body	130	37.3	61	36.5	69	
Antrum	187	56.3	89	51.9	98	
Local invasion**						< .001
T1	44	2.9	6	17.4	38	
T2	76	17.9	37	17.8	39	
T3	294	78.7	163	59.7	131	
T4	12	0.5	1	5.1	11	
Lymph node involvement***						= .624
N0	181	41.1	85	43.8	96	
N1	141	36.2	75	30.1	66	
N2	95	22.7	47	21.9	48	
N3	9	0.0	0	4.1	9	
Stage****						= .562
IA	34	0.5	1	15.0	33	
IB	58	15.5	32	11.9	26	
II	116	31.4	65	23.3	51	
IIIA	118	30.9	64	24.7	54	
IIIB	83	21.3	44	17.8	39	
IV	17	0.5	1	7.3	16	

* ; Not specified in 77 patients (47 in PT1 and 30 in PT2)

### Surgery

Treatment characteristics are shown in Table 2. All patients underwent subtotal or total gastrectomy: 44%/56% for TP2 patients, and 55%/45% for TP1 patients respectively. Twenty-nine splenectomies were performed, four of them with subtotal gastrectomy and twenty-five with total gastrectomy. More extended lymph node dissections were performed among the TP2 patients compared with those PT1 patients (D2 65% vs. 10%, respectively).

**Table 2:** Surgical Treatment and Adjuvant Chemotherapy Details

		**Year of diagnostic**				
			
		**Before 1990**		**1990 and after**		
			
	**Total**	**%**	**n**	**%**	**n**	**ρ **
**Gastrectomy**						= .055
**Subtotal**	168	55.3	83	44.3	85	
**Total**	175	44.7	68	55.7	107	
Splenectomy*	29	3.4	5	12.5	24	
Lymphadenectomy**						< .001
**D1**	249	89.3	184	34.6	65	
**D2**	145	10.7	22	65.4	123	
**Adjuvant Chemotherapy**						= .436
**No**	192	47.3	98	42.7	94	
**Yes**	235	52.7	109	57.3	126	
**Chemotherapy schedule**						< .001
**MMC**	133	81.7	89	34.9	44	
**MMC-TG**	102	18.3	20	65.1	82	

*; Not specified in 54 patients (47 in PT1 and 30 in PT2)

** Not specified in 32 patients (31 in PT1 and 1 in PT2)

Perioperative mortality in TP2 patients was 4% (9 out of 219), mainly by sepsis or pulmonary thromboembolism, and 1% (2 out of 207) (p = 0.091) in TP1 patients.

Forty-five percent of the patients received no adjuvant treatment after surgery, followed by clinical checking until progression or death. No difference was observed in the rate of patients that received no adjuvant chemotherapy between both TP2/TP1 periods (42.7% versus 47.3%; p = .436). Among those patients who received adjuvant chemotherapy, the MMC-TG combination was the most frequently prescribed regimen in TP2 patients (65%) compared with MMC alone, the most commonly used treatment in TP1 patients (82%).

### Survival analysis

**Figure 1:**
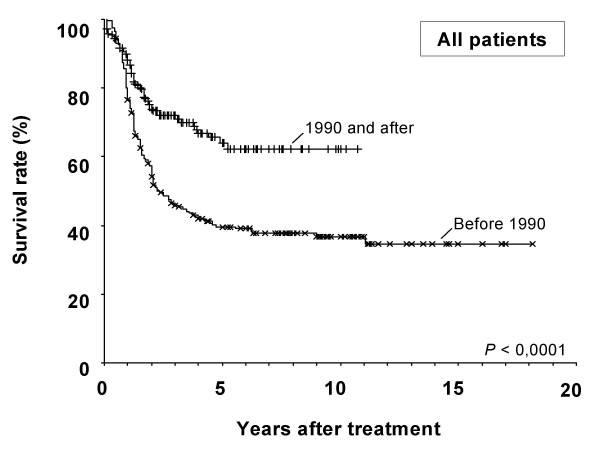
Kaplan-Meier survival curves of all 426 gastric cancer patients. Patients treated in1990 and after had a significantly better long-term survival than those treated before that year (median survival of >120 m versus 28 m; p < 0.0001).

Median follow-up for all patients was 216 months. At the time of analysis, the 219 TP2 patients had a median follow up of 96 months, whereas the 207 TP1 patients had been followed-up for a median of 260 months. Median survival was 120+ months (median not reached) and 32.31 months for each period, respectively. 5-year overall survival was 66% for TP2 patients versus 42% for TP1 patients (p < 0.0001). Figure 1 shows a Kaplan-Meier survival curves with *plateau *beginning in 5^th ^year in both groups, and maintains this line for more than 10 years.

### Staging

**Figure 2:**
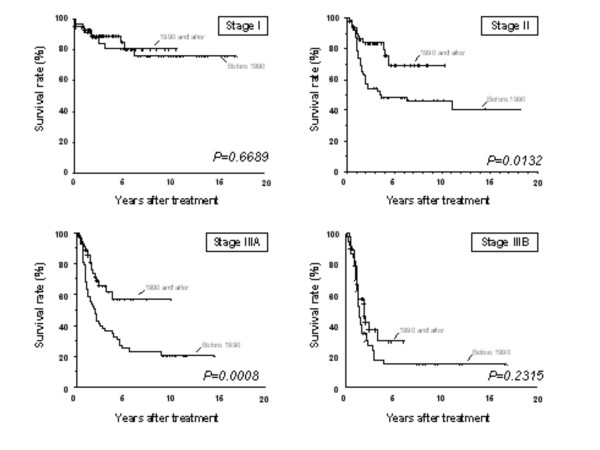
Kaplan-Meier survival curves of patients at stages I, II, IIIA and IIIB. Patients with stages II and IIIA treated in 1990 and after had a significantly better long-term survival than those treated before (median survival of >120 m versus 78 m; p = 0.0132 and >120 m versus 23 m; p = 0.0008, respectively)

**Figure 3:**
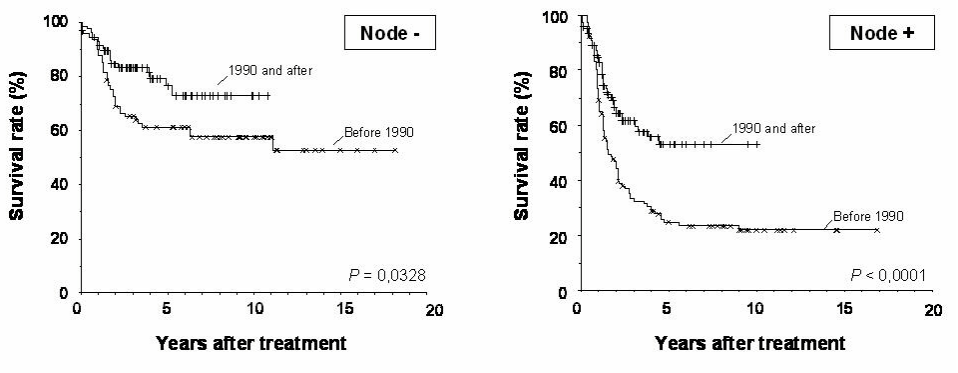
Kaplan-Meier survival curves of patients according to nodal involvement. Significantly better survival was observed among patients treated after 1990, independently of nodal involvement (5-y overall survival of 52% versus 23%; p = 0.0001 and 73% versus 62%; p = 0.0328, respectively)

When subgroups analysis was done according to the staging, the significant improvement in survival seems to be restricted to stages II and IIIA, with an insignificant improvement at stage IIIB (see table 3). Five-year overall survival at stages II, IIIA and IIIB for each TP2/TP1 period was 70% vs. 51% (p = 0.0132), 57 vs. 23% (p = 0.0008) and 30 vs. 15% (p = 0.2315), respectively (Figure 2). Both node negative and positive patients show better survival in TP2 patients compared with those treated before 1990 (5-year overall survival: 73% versus 62%, p = 0.0328 and 53 versus 23%, p < 0.0001) (Figure 3). An improvement in survival has been observed in D2 lymphadenectomy patients comparing TP2 with TP1 (5-year overall survival: 66% versus 42%, p = 0.0076) as patients treated with adjuvant chemotherapy as patients non treated with adjuvant chemotherapy.

**Table 3:** Comparison of median survival and 5-year survival rates between subgroups of patients according to the period of treatment

	**Year of diagnostic**						
		
	**Before 1990 (n = 207)**			**1990 and after (n = 219)**			
		
	**n**	**x OS (m)**	**5-y OS (%)**	**n**	**x OS (m)**	**5-y OS (%)**	**ρ **
**Gender**							
**Male**	135	26	32	141	120+	58	< .0001
**Female**	72	113	53	78	120+	69	= .0434
**Age, years**							
**< 61**	112	34	42	69	120+	57	= .0504
**> 61**	95	31	37	149	120+	64	< .0001
**Tumor location**							
**Cardia**	10	36	26	22	120+	90	= .0021
**Body**	59	33	38	69	120+	62	= .0022
**Antrum**	89	38	44	98	120+	59	= .0253
**Lymph node involvement**							
**N0**	85	216+	62	96	120+	73	= .0328
**N+**	122	22	23	123	120+	52	< .0001
**Stage**							
**I**	33	204+	81	59	120+	81	= .6689
**II**	65	78	51	51	120+	70	= .0132
**IIIA**	64	23	23	54	120+	57	= .0008
**IIIB**	44	19	15	39	23	30	= .2315
**Lymphadenectomy**							
**D1**	184	31	39	65	26	26	= .6718
**D2**	22	52	42	123	120	66	= .0076
**Adjuvant Chemotherapy**							
**No**	98	22	30	94	60	45	= .006
**Yes**	109	58	48	126	120+	73	= .0003
**Chemotherapy**							
**MMC**	89	48	47	44	120+	58	= .2346
**MMC-TG**	20	80	54	82	120+	87	= .0113

### Survival and adjuvant chemotherapy

When comparing the outcome of patients treated with adjuvant chemotherapy using MMC only with those patients treated with adjuvant MMC-TG during period TP2, a better global survival was observed in patients treated with the two-drug regimen. Global survival at 5 years for those treated with adjuvant MMC was 58% for TP2 patients, and 47% for those TP1 patients but these differences were not statistically significant (p = 0.2346.) Nevertheless, for those TP2 patients treated with adjuvant MMC-TG, survival at 5 years was 87% while for TP1 patients the rate was 54% (p = 0.0113). MMC-TG was the adjuvant chemotherapy in 44% of the patients after 1995 versus 20 % of the patients between 1990 and 1994.

### Timing of treatment

When we look at the date when patients were diagnosed and treated, if we divided in five-year period, we can see that there is an improvement in survival in patients treated more recently. The best results have been mainly observed in patients treated after 1995 compared with patients treated earlier with a rate of long term survivors of 73% and 41% respectively (p < 0.0001).

**Figure 4:**
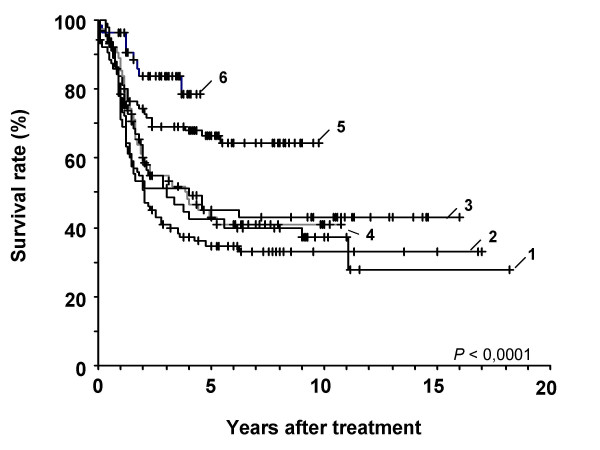
Kaplan-Meier survival curves of patients divided in 5-years periods according to the date of diagnosis: 1 = 1974–1979 (n = 46); 2 = 1980–1984 (n = 94); 3 = 1985–1989 (n = 67); 4 = 1990–1994 (n = 76); 5 = 1995–1999 (n = 88); 6 = 2000–2002 (n = 55). Statistically significant improvement of survival was shown in periods 5 (1995–99) and 6 (2000–02) when compared with period 4 (1990–94) (p = 0.0006). No differences were observed between survival of patients in period 4 (1990–94) and periods previous to year 1990.

The 87 patients treated between 1995 and 1999 have an average follow-up of 90 months. Survival at 5 years is 67%. The 55 patients treated since 2000 have a follow-up of 48 months and of these, 9 have died of the disease (16%) and 4 more are alive but have been relapsed (7%) (Figure 4).

The Cox multivariate analysis applied using the significant variables in the univariate analysis showed that stage (p = 0.002) and period (TP2 over TP1) (p < 0.001) of treatment continue to be statistically significant as independent variables. When we analyzed the period of treatment before and after 1995 (instead of 1990) the stage (p = 0.003) and period of treatment (p < 0.001) were also independent variables.

## Discussion

Gastric cancer can be a fatal disease even in its earliest stages. There is a survival rate of approximately 40% at 5 years in patients with loco-regional disease. These patients would be candidates for procedures such as gastrectomies and lymphadenectomies. Nevertheless, as mentioned before, an improvement in cure rates has been observed in recent years. In some cases this improvement is produced through surgery plus chemotherapy or radiotherapy but in other cases, through expert surgery only. Patients with affected lymph nodes in the postoperative pathological staging have the worst prognosis. In our series, survival at 5 years in patients with positive nodes rose from 23% to 53% on the basis of whether they were treated before or after 1990. These results are superior to those published by Sasako *et al *[20], which were 30%–40%. This data suggests that since 1990, there has been improved postoperative staging and in contrast, prior to 1990, there may have been patients who were down-staged.

When we compare patients treated after 1990, survival rate at 5 years according with the stages, we can seen that our results are similar to those published by Japanese groups and both are better that those published by other Western authors (Table 4).

**Table 4:** Five-year survival rates of curatively resected gastric cancer patients in Japan, Western patients** and our series (patients treated after 1990)

Stage*	**Western**	**Japanese**	**Grau *et al***
**Ia**	92	96	81
**Ib**	84	85	
**II**	53	72	70
**IIIa**	32	49	57
**IIIb**	9	30	30

*UICC-TNM stage.

** Data from ref. 9

Our results indicate an improvement in global survival in patients treated after 1990 when D2 dissection and adjuvant chemotherapy was the treatment applied in the majority of patients. However, this improvement in survival is clearer still from 1995 onwards. The reasons for this improvement cannot be explained simply by earlier diagnosis in patients with earlier stage as it occurs in different stages of the disease. Only stage IIIB does not show a significant statistical improvement in the survival curve, probably because of the low number of patients of our series in this situation. Better post-operative care was not the explanation for this improvement, as perioperative mortality was higher in patients treated after 1990 than before, probably due to the greater number of D2 lymphadenectomy carried out. This procedure shows mortality rates higher than those of D0 and D1 dissections in the majority of studies [21]. In addition, the majority of surgical procedures were carried out by the same team of 4 surgeons who have acquired wider experience through the years. There are evidences to show that the surgeon's learning curve can improve the final results of treatment [22] and partially explain the survival improvement after 1995.

Adjuvant chemotherapy with 2 drugs has shown itself to be superior to that with MMC only [23]. Also, D2 surgery along with chemotherapy with 2 drugs has demonstrated long-term survival rates of up to 75%. All of this data suggested that improved outcomes could be the result of better surgery carried out by more experienced surgeons together with adjuvant chemotherapy based on MMC plus Tegafur.

Several factors could produce a time-related bias in the studies focused on temporary improvements in survival of diseases. These include changes in radiological facilities for detection and diagnosis of metastases, changes in diagnostic criteria and evolution of clinical characteristics, allowing a more accurate staging of the patients. Nevertheless, in our study, the radiological facilities used to detect metastases of primary gastric cancer were similar after 1990 and include liver ultrasound, chest X-rays, and abdominal CT scan for confirmation.

One can argue that the improvement is related to poor results in the first cohort. Nevertheless, the survival rates observed during this time period (1975 to 1989) are not different from those reported in other hospital-based cohort studies [24]. Our study was not designed to identify what intervention led to survival improvements. Nevertheless, several hypotheses could be put forward to explain the improvements in survival observed in our study. The first hypothesis is that advances in surgical resection have led to improvements in survival. The study by Bonenkamp *et al *[9] was unable to establish significant differences between D1 and D2 resection. However, one explanation would be that the D1 control group had longer than expected survival due to the action of an experienced surgeon. If macroscopically visible lymph nodes were found in the mesenteric region during a surgical intervention, the original objective of which was a D1 resection, we must suppose that the resection proceeded with a wider intervention to include those nodes and therefore became a D2-like dissection.

As in other retrospective analysis [25], our study did not assess which one of these surgical options has changed the outcome of patients. Nevertheless, the predominant use of adjuvant chemotherapy after D2 dissection showed the best results. Post-surgical radiation therapy was not included in the adjuvant treatment of these patients.

Recent cure rates should be reconsidered as a point of reference for studies that are currently working with resectable gastric cancer [26]. On the other hand, our data suggest that timing is an important prognostic factor when treating patients with gastric cancer, probably through improving the application of multidisciplinary treatment. On the light of this retrospective analysis we may suggest that Japanese-style gastrectomy with D2 lymphadenectomy carried out by expert surgeons, followed by adjuvant chemotherapy with MMC-TG can be a good option for treating Western patients with locally advanced gastric cancer.

## Authors disclosures of potential conflicts of interest

The author(s) declare that they have no competing interest.

## Authors' contributions

**JJG **designed the study, collected the data and wrote the first draft of the manuscript.

**RP, MM, JDD **all contributed to collected data and the preparation of the first draft of the manuscript.

**MMZ **participated in the design of the study and in drafting and revising the manuscript

**JF, OV, CF **and **JCG **all contributed to patient recruitment and revising the manuscript.

All authors read and approved of the manuscript.
